# p16^INK4A^ and p14^ARF^ Gene Promoter Hypermethylation as Prognostic Biomarker in Oral and Oropharyngeal Squamous Cell Carcinoma: A Review

**DOI:** 10.1155/2014/260549

**Published:** 2014-04-07

**Authors:** A. Al-Kaabi, L. W. van Bockel, A. J. Pothen, S. M. Willems

**Affiliations:** ^1^Department of Pathology, University Medical Centre Utrecht, P.O. Box 85500, 3508 GA Utrecht, The Netherlands; ^2^Department of Radiation Oncology, University Medical Centre Utrecht, P.O. Box 85500, 3508 GA Utrecht, The Netherlands; ^3^Department of Otorhinolaryngology, University Medical Centre Utrecht, P.O. Box 85500, 3508 GA Utrecht, The Netherlands

## Abstract

Head and neck squamous cell carcinoma is a heterogeneous group of tumors with each subtype having a distinct histopathological and molecular profile. Most tumors share, to some extent, the same multistep carcinogenic pathways, which include a wide variety of genetic and epigenetic changes. Epigenetic alterations represent all changes in gene expression patterns that do not alter the actual DNA sequence. Recently, it has become clear that silencing of cancer related genes is not exclusively a result of genetic changes such as mutations or deletions, but it can also be regulated on epigenetic level, mostly by means of gene promoter hypermethylation. Results from recent studies have demonstrated that DNA methylation patterns contain tumor-type-specific signatures, which could serve as biomarkers for clinical outcome in the near future. The topic of this review discusses gene promoter hypermethylation in oral and oropharyngeal squamous cell carcinoma (OSCC). The main objective is to analyse the available data on gene promoter hypermethylation of the cell cycle regulatory proteins p16^INK4A^ and p14^ARF^ and to investigate their clinical significance as novel biomarkers in OSCC. Hypermethylation of both genes seems to possess predictive properties for several clinicopathological outcomes. We conclude that the methylation status of p16^INK4A^ is definitely a promising candidate biomarker for predicting clinical outcome of OSCC, especially for recurrence-free survival.

## 1. Introduction

Head and neck cancer is one of the most prevalent malignancies and causes a significant burden of morbidity and mortality each year, accounting for over half a million new cases worldwide, mostly men [1]. Traditionally this cancer encloses a wide variety of malignant tumors with squamous cell carcinoma (SCC) being, by far, the most common subtype. Each subtype is further subdivided according to anatomical location in the head and neck area, for example, oral cavity, epipharynx, oropharynx, larynx and hypopharynx, and different histopathological features [2, 3].

Tobacco and alcohol consumption are major aetiological risk factors in head and neck squamous cell carcinoma (HNSCC) [4, 5]. These two factors contribute to the development of HNSCC, especially affecting men in advanced age and women due to increasing smoking rates among female gender during the past decades. In recent years, human papillomavirus (HPV) infection is also recognized as an important determinant for oropharyngeal cancer in a new group of nonsmoking, nondrinking younger adults who have shown increased incidence of this cancer [6, 7]. Other predisposing factors for HNSCC are exposure to radiation or environmental toxins, betel nut chewing, and immunosuppression [8].

HNSCCs have in common that they are preceded by precancerous lesions [9] and share, to some extent, the same multistep carcinogenic pathways, subsequently leading to invasive squamous cell carcinoma [3]. This multistep process includes a wide variety of genetic changes. In the past decade, it has become clear that silencing of tumor suppressor genes, one of the main principles of carcinogenesis, is not exclusively a result of genetic changes such as mutations or deletions, but can also be regulated on epigenetic level [10–12].

In contrast to genetic DNA alterations, epigenetics encompass all changes in gene expression patterns that do not alter the actual DNA sequence. One of the most extensively studied epigenetic modifications is gene promoter hypermethylation, which usually results in transcriptional inactivation of the gene. More interesting, gene silencing in cancer might occur more frequently by means of promoter hypermethylation than by DNA mutations [13]. Unlike genetic alterations (such as gene mutation or deletion), DNA hypermethylation is much more dynamic and often reversible in nature [11, 14] and is, therefore, an attractive target for new therapeutic agents.

Considering HNSCC as a highly inhomogeneous collection of tumors regarding their aetiology, histology, clinical course, and prognosis, it is quite appealing to investigate whether this heterogeneity can be attributed to differences in molecular basis, especially variations in DNA hypermethylation [3, 15]. Therefore, identification of different (epi)genetic profiles will allow for a new classification of HNSCC into molecular subtypes. Moreover, a distinct epigenetic profile, also referred to as “epigenome,” is interesting as it offers new opportunities to develop new specific biomarkers for each subtype and with that improving screening, early diagnosis, and therapeutic decision making [16].

We have chosen oral and oropharyngeal squamous cell carcinoma (OSCC) as the main focus of this review, because of the alarming increase in the incidence rates of this specific subtype [6]. Furthermore, the need for novel, more specific biomarkers in HNSCC is endorsed by the fact that, despite rapid advances in the sphere of diagnosis and therapy, the prognosis, in particular for HPV negative OSCC, has not been improved in the past decades. The prognosis remains unchanged at a five-year survival of 50% [17].

Here, we briefly review some promising epigenetic factors in OSCC. The main emphasis will be on promoter hypermethylation of the cell cycle regulatory proteins p16^INK4A^ and p14^ARF^, both encoded by the CDKN2A gene, one of the most widely investigated genes in HNSCC. The aim of this review is to address the clinical significance of p16^INK4A^ (a.k.a. p16) and p14^ARF^ (a.k.a. p14) hypermethylation and whether they can serve as prognostic biomarkers in OSCC.

## 2. Brief Introduction into Gene Promoter Hypermethylation

Epigenetics mostly refers to promoter hypermethylation, next to other alterations such as histone deacetylation, global genomic hypomethylation, and histone remodelling. DNA hypermethylation represents the covalent addition of a methyl group to a cytosine nucleotide resulting in 5-methylcytosine. This modification is catalysed by the DNA methyltransferase enzyme family (DNMTs) with S-adenosyl-methionine acting as a methyl donor [11]. Cytosine methylation occurs in CpG dinucleotides that have an asymmetrical distribution throughout the genome. However, a small proportion of the CpG dinucleotides are clustered together in 500 base pair long regions, called “CpG-islands,” where they take up more than half of the nucleotides. These CpG-islands are known to be located in promoter regions of approximately 50% of mammalian genes [18].

The promoter sequence is a gene control region where general transcription factors and RNA polymerases bind, before DNA transcription is initiated. Deletions, mutations, and promoter hypermethylation are important mechanisms which can alter gene activity. According to Knudson's two-hit hypothesis, a tumor suppressor gene is silenced when both alleles are inactivated. After a mutational first hit of one allele, promoter hypermethylation can silence the second normal allele without introducing changes into the DNA sequence. In sporadic cancer, two point mutations are rarely responsible for biallelic inactivation of tumor suppressor genes, while promoter hypermethylation of both alleles is more common [11, 19].

Under physiological conditions, there is a basic genomic methylation pattern, sometimes referred to as “methylotype” [20]. In the basic pattern the majority of genes have promoter regions with nonmethylated CpG-islands, whereas methylation of CpG dinucleotides outside these CpG-islands is abundantly present. This is thought to be part of a natural defence mechanism which safeguards the integrity of the genome during replication: on one hand by imposing transcriptional repression on large parts of mainly noncoding DNA, which may contain harmful sequences, and on the other hand allowing transcription of coding DNA through gene promoter hypomethylation [3].

In neoplastic cells, however, there is a significant shift in the basic pattern of DNA methylation characterized by increased CpG methylation in promoter regions of specific genes, mainly involved in DNA repair, apoptosis, cell cycle regulation, and tumor suppression. Simultaneously, loss of methylation in otherwise silenced regions takes place, a process named “global hypomethylation,” with increased overall gene expression level due to weakened transcriptional repression. These changes indeed affect genetic stability and contribute to cancerization of the cell. Aberrant promoter hypermethylation has been observed is almost all types of cancer, including HNSCC, and the pattern of methylation appears to be tumor type specific [16, 21]. In addition to that, the possible reversibility of abnormal methylation makes DNA hypermethylation an appealing target for new cancer-specific therapy. In fact, several chemotherapeutic agents have been found to possess demethylating properties which can reverse the transcriptional silencing of genes [22].

## 3. Promoter Hypermethylation in Oral and Oropharyngeal Squamous Cell Carcinoma (OSCC)

Numerous studies have investigated epigenetic alterations in OSCC and have found that promoter hypermethylation of multiple genes is highly prevalent. The silenced genes are typically tumor suppressor genes.  Table 1 is a summary of sixteen genes with substantial evidence for hypermethylated promoter region in OSCC; also their reported clinicopathological associations are summarized. The inclusion criterion was hypermethylation, proven in more than one study. Furthermore, we selected and classified candidate genes according to their potential biomarker application as indicated by reported associations (Table 2). The main emphasis of this review is on the p16^INK4A^ tumor suppressor gene because its role has been well studied in oral and oropharyngeal squamous cell carcinoma (Table 1). We also discuss the hypermethylation of the p14^ARF^ tumor suppressor gene, which is associated with a more favourable prognosis in OSCC.

**Figure 1:**
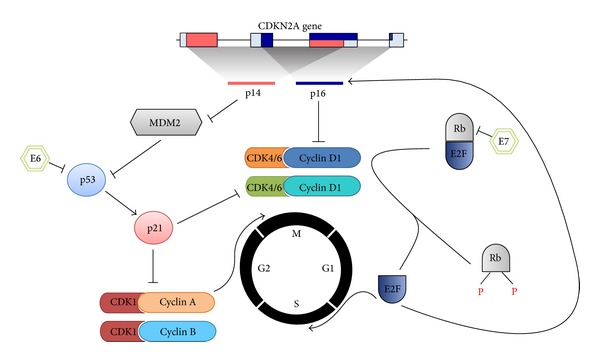
Cell cycle arrest by CDKN2A. The CDKN2A gene encodes two alternatively spliced transcripts, p16^INK4A^ and p14^ARF^, which differ in their first exon. The p16^INK4A^ protein inhibits the CDK4/6-cyclin D1 complexes, keeping the retinoblastoma (Rb) proteins in a dephosphorylated state, and enables binding and inactivating the E2F transcription factors. Free E2F ensures the transcription of various proteins, most of them are necessary for progression to S phase. P16^INK4A^ is also upregulated by E2F. In contrast, p14^ARF^ stabilizes and thus activates the tumor suppressor gene p53 by inhibiting MDM2, which inactivates p53 by ubiquitin-mediated degradation. Active p53 induces the expression of p21, a negative cell cycle regulator which is an inhibitor of the CDK1-cyclin A/B complexes, thereby preventing the progression from G2 phase to metaphase. The human papillomavirus oncoproteins E6 and E7 interfere in the Rb pathway and in the p53 pathway, in order to bypass the cell cycle checkpoints. The E7 oncoprotein promotes the progression to S phase. It binds the Rb proteins and thereby releases the E2F transcription factors. The E6 protein targets p53 and induces loss of function by degradation.

The proteins p16 and p14 are two alternative splice variants of the CDKN2A gene, located on chromosome 9p21. Both proteins function as inhibitors of cell cycle progression (Figure 1). The p16 protein promotes senescence and differentiation by interfering in the retinoblastoma (Rb) pathway. It prevents entry into S phase by inhibiting the CDK4/6-cyclin D1 complexes, thereby preventing the phosphorylation of Rb proteins. As a result, E2F transcription factors are inactivated, as the Rb-E2F complex remains intact. The p14 protein activates the tumor suppressor gene p53 by inhibiting MDM2, an ubiquitin ligase that marks p53 for degradation. which in turn leads to cell cycle arrest or apoptosis in cells [3, 23].

In the last decade, aberrant promoter hypermethylation of p16 and p14 has been observed in oral and oropharyngeal cancer tissue (Table 1) as well as premalignant oral lesions [24–28] and histologically healthy mucosa surrounding the tumor [29–31]. More important, p16 and p14 gene inactivation is primarily due to promoter hypermethylation [32]. In one of the earliest studies focussing on OSCC as a distinct entity, Wu et al. demonstrated that the vast majority of the tumors (>80%) had loss of p16 expression [33]. Interestingly, p16 promoter hypermethylation appeared to be more common than point mutation (23% and 7%, resp.). In a Brazilian cohort of 45 patients with resected primary OSCC tumors, the methylation status of four genes was investigated and high rates of hypermethylation for CDKN2A (p16 and p14), EDNRB, RUNX3, and SFN were found [34]. They also reported more CDKN2A and EDNRB promoter region hypermethylation in subjects with lymph node metastases [34]. In an Indian cohort, four genes were selected and their methylation status was evaluated in a sample of 92 OSCC patients [30]. The promoter regions of EDNRB, KIF1A, p16, and DCC were found to be highly methylated in tumor tissue, and p16 methylation was associated with nodal involvement.

In another study, a semiquantitative approach (pyrosequencing) was adopted in order to quantify the promoter hypermethylation of five genes in OSCC samples. The association between the quantitative methylation index and clinicopathological variables was analysed [35]. No such association was observed. However, the methylation of the genes p16, CYGB, and CYCA1 was highly tumor specific because clear resection margins contained significantly less abnormal methylation for these genes. Also, hypermethylation of ECAD and RAR*β* was observed in tumor tissue and adjacent healthy mucosa. No significant hypermethylation was observed in healthy control tissue [30, 35].

Several studies have evaluated the presence of promoter region hypermethylation in oral premalignant lesions. In an early study, loss of p16 function was reported in a small number of patients (17/37) with leukoplakia, accounting for 5 out of 8 patients who developed malignant transformation [24]. Also, increasing p16 gene promoter hypermethylation rates were reported for mild to severe dysplastic lesions, 30% and 82%, respectively [25]. In patients with severe dysplastic epithelial lesions, Kresty et al. detected a p16 methylation rate of 57%, while p14 was methylated in 3.8% of the samples [26]. An association was found for p16 hypermethylation with loss of heterozygosity and lesions of the tongue and floor of the mouth.

Two studies investigated the prognostic significance of p16 hypermethylation in oral epithelial dysplasia [27, 28]. In the first study, a significant proportion of patients with malignant transformation of epithelial dysplasia had p16 hypermethylation compared to patients with no malignant transformation (57% versus 8%, *P* = 0.002). However, p16 did not correlate with time of onset of transformation [27]. The other study supported these findings, describing a significantly higher progression rate for oral dysplasia to OSCC in p16 hypermethylated cases (43.8% versus 17.4%; OR = 3.7) [28]. This effect was more evident in patients aged above 60 years (OR = 12.0, *P* = 0.013) and subjects with moderate epithelial dysplasia (OR = 15.6, *P* = 0.022). These findings suggest that p16 hypermethylation is a powerful marker for selecting patients with precancerous lesions who are at risk for progression to malignant disease.

Although not fully convincing due to small sample size, the feasibility of p16 hypermethylation in surgical resection margins as a prognostic factor was investigated by Goldenberg and colleagues demonstrating hypermethylation in margins of three patients (3/13) with SCC of the tongue [36], and in a later study, positive margins were reported in 4 OSCC patients, of which two developed a local recurrence [37]. Recently, a prospective study including a larger number of Indian patients with SCC of the tongue showed that 43% (13/30) of histologically tumor-free margins contained p16 hypermethylation and further analysis showed a 6.3-fold increased risk for local recurrence for these 13 patients [31]. Still, the p16 methylation status did not affect the overall survival rate. Also a more recent study in OSCC resection margins could not establish a correlation between p16 hypermethylation and overall survival [38]. To our knowledge, these two studies are the first to evaluate the significance of p16 hypermethylation as a predictive and prognostic marker in surgical resection margins. Further research is needed to investigate whether intraoperative p16 hypermethylation analysis in surgical margins results in more accurate resection with less recurrence compared to the conventional histopathological assessment.

The fact that aberrant promoter hypermethylation of p16 and p14 is detected in both peritumoral tissue and premalignant lesions suggests that these epigenetic alterations are an early event making tissue more prone to neoplastic transformation. These findings are in concordance with the concept of “field cancerization,” originally proposed by Slaughter et al. in 1953 to explain the high recurrence rates of head and neck cancer [39]. They hypothesized that multiple acquired genetic defects in large patches of mucosa in the upper aerodigestive tract make morphologically normal epithelium prone to dysplastic or malignant transformation [3, 39]. This so-called “fields” are not limited to the boundaries of the malignancy but extend into surgical resection margins and increase the risk of local relapse or a second primary tumor.

## 4. Promoter Hypermethylation and Clinicopathological Associations

Many studies have described correlations between p16 and p14 promoter hypermethylation and different clinical outcomes in OSCC (Table 1). In the study of Sailasree et al., both p14 and p16 hypermethylation was investigated in respect to outcome in OSCC. A significant association with favourable outcome was found in OSCC with p14 hypermethylation, whereas p16 hypermethylation is associated with poor survival [40]. However, reports on other clinicopathological associations are more inconsistent. In a cohort of 96 OSCCs aberrant methylation of p16 and p14 was observed in 29% and 14% of the tumors, respectively [41]. Younger age and tumor size (lower T stage) were significantly associated with p16 hypermethylation; remarkably p14 hypermethylation was significantly associated with a longer overall survival time. Similar results by Ishida et al. related hypermethylation of p14 to tobacco and alcohol consumption, increased lymph node invasion, and higher clinical stage [42]. p16 hypermethylation correlated with increased tumor size and higher clinical stage, although association did not reach significance. In another study, these findings were verified in an Indian cohort of 116 OSCC patients [40]. They found that cases with hypermethylation of p16 had a threefold higher risk for disease recurrence (RR = 3.3), whereas p14 methylation was significantly associated with reduced recurrence rate (RR = 0.109). Hypermethylation of both markers did not show any correlation with overall survival rates; yet, high p16 protein expression was associated with reduced residual disease after treatment (RR = 0.351) and increased overall survival during followup (RR = 0.318). More recently, a study in buccal SCC, one of the most frequent types of OSCC, indicated a relation between p16 hypermethylation and lymph node metastasis and poor overall survival [43]. However, in concordance with previous studies, such relation did not reach level of significance in multivariate analysis.

To our knowledge, there are few studies that address a possible link between p16 or p14 promoter hypermethylation and HPV infection in OSCC. In a small cohort of 24 oral and oropharyngeal SCC samples, HPV 16 positive tumors seemed to correlate with p16 overexpression, but not with p16 hypermethylation [44]. This association was also absent in a Brazilian cohort predominated by SCC of the oral cavity (90%) [34]. However, one recent case-control study found a significantly higher prevalence rate (69.2%) for p16 hypermethylation in HPV 16 infected oral epithelial dysplasia samples, compared to noninfected samples (20.8%) [45]. DNMT1 and DNMT3b levels did not correlate with HPV status or p16 hypermethylation. Since HPV positive tumors are more likely to overexpress p16, the authors hypothesize that the observed association between HPV 16 and p16 hypermethylation could explain the unusual low p16 protein expression in a subset of HPV positive tumors with less favourable prognosis. More clinical evidence is needed to back up this hypothesis. For future research, it would be appealing to investigate the molecular impact of HPV infection on epigenetic regulation, with particular attention to p16 promoter hypermethylation, and if there is a modifying relation to elucidate the molecular advantages for HPV in adopting a mechanism that downregulates p16.

Thus, the available data from recent studies in OSCC carefully suggest that promoter hypermethylation of p16 and p14 is tumor specific, since transcriptional silencing of both genes by hypermethylation is highly prevalent in tumor tissue and rather lacking in healthy controls. Based on reported clinical associations, we conclude that there is sufficient evidence in OSCC for p16 hypermethylation as a predictive marker for a less favourable clinical outcome. Vice versa, high p16 expression levels, proven by immunohistochemistry, are associated with improved prognosis [46], which supports our previous conclusion. However, clinical impact of p16 hypermethylation on overall survival remains inconclusive. Therefore, we acknowledge the potential application of p16 hypermethylation as a biomarker for recurrence in OSCC. There is a need for larger survival studies in order to overcome the current inconsistency in the literature and to investigate whether p16 hypermethylation is applicable as a fully independent prognostic marker. First results regarding p14 hypermethylation are promising; still, the available evidence on OSCC is too limited to draw conclusions. Aberrant p14 hypermethylation certainly deserves more attention and hopefully more research will be dedicated to the clinical significance of this tumor suppressor gene.

## 5. Conclusion

Thanks to advances in the field of epigenetics, our understanding of the molecular origin of cancer has changed rapidly. There is now sufficient and well-established evidence that epigenetic DNA alterations play a decisive role in the development of cancer by regulating the transcription of many (tumor suppressor) genes. Furthermore, it has become clear that in many tumor types specific epigenetic features can be distinguished and that DNA hypermethylation is a major determinant of the “epigenome.” The key question remains whether extended knowledge of cancer epigenetics will result in a new molecular classification of this disease in well-defined and more uniform subcategories.

In this review we have focussed on oral and oropharyngeal squamous cell carcinoma as subcategories of HNSCC, which are likely to have their own distinct epigenetic profile. How these differences do occur is not clear, but they might be explained by the alternative aetiologies of head and neck tumors since risk factor exposure is different for age, ethnicity, and geographic location.

Concerning the objective of this review, to address the feasibility of aberrant promoter hypermethylation of p16 and p14 as biomarkers in OSCC, one can draw several conclusions based on the available reports. First, aberrant hypermethylation of p16 and p14 is a cancer-specific finding, since it is significantly observed in OSCC and is not likely to be found in healthy control tissue. Secondly, promoter hypermethylation of p16 and p14 occurs early in the process of cancerization, as both are detected in oral precancerous lesions as well as peritumoral tissue. Last but not least, a growing body of evidence has confirmed the predictive value of p16 promoter hypermethylation for several clinicopathological parameters, including progression of premalignant lesions to OSCC, advanced disease, local recurrence, and disease-specific survival. The methylation status of p16 is definitely interesting as a candidate biomarker for predicting the clinical course of OSCC. However, more prospective studies are needed to affirm the clinical applicability of p16 hypermethylation in larger groups of patients. Early assessment of p16 hypermethylation might enable the identification of subgroups of patients with poor prognosis, who might require a different therapeutic approach. Therefore, we recommend future research to explore the position of this biomarker in the clinical management of OSCC and to evaluate whether it can contribute to personalised treatment strategies.

## Figures and Tables

**Table 1:** Candidate genes frequently silenced by promoter hypermethylation in OSCC tumor tissue.

Mechanism	Gene	Gene function	Clinicopathological association^a^	References
Cell cycle regulation	CYCA1	Cell cycle	Lower histological grade	[37, 47]
	CHFR	Early G2/M checkpoint	Higher T status	[48, 49]
	p14^ARF^	Proapoptosis	LNM^b^, T status (T2-3), advanced stage Reduced recurrence rate, favourable prognosis	[32, 40–42, 50, 51]
	p15	Cyclin-dependent kinase inhibitor 2B	Anatomic site (tongue SCC) Alcohol and tobacco use	[35, 41, 50]
	p16^INK4A^	Regulates cell cycle G1 progression	Larger tumor size, LNM, advanced stage Younger age, increased recurrence rate, poor prognosis	[29, 30, 32–35, 37, 40–42, 50, 52–54]

DNA repair	hMSH1/hMSH2	DNA mismatch repair	—	[35, 38, 55]
	MGMT	Guanine alkylation repair	Reduced overall survival Reduced disease-free survival	[29, 35, 38, 56]

Signal transduction	EDNRB	Endothelin receptor type B	Alcohol and tobacco use	[30, 34]
	RUNX3	Wnt pathway antagonist	LNM, advanced stage, poor differentiation	[34, 38, 57, 58]
	SFRP1	Wnt pathway antagonist	Male gender	[59]

Tissue invasion/metastasis	ECAD	Calcium-dependent cell-cell adhesion glycoprotein	LNM, increased metastatic potential Reduced disease-free survival	[35, 60–62]

Tumor suppression	HIN1	Inhibitor Ras pathway	Reduced disease-free survival	[63]
	DAPK1	Proapoptosis	LNM	[38, 41]
	DCC	Proapoptosis	Invasion of bone and deep tongue Reduced survival	[30, 41]
	RASSF1A/RASSF2	Negative RAS effector, proapoptotic, microtubule stabilization	Decreased disease-free survival radioresistance	[38, 63]

Other	KIF1A	Cell division and microtubule-dependent intracellular organelle transport	Malignant histology	[30, 64]

^a^Reported significant associations and trends.

^b^Lymph node metastasis.

**Table 2:** Classification of hypermethylated candidate genes in OSCC according to their potential biomarker application.

Biomarker type	Genes
Diagnostic	CYCA1, EDNRB, KIF1A, RUNX3
Prognostic	p14, p16, MGMT, ECAD, DCC, DAPK1
Predictive	p16, RASSF
Screening	p15, EDNRB
